# Supplementary material for: Structure‐function analysis of the maize bulliform cell cuticle and its potential role in dehydration and leaf rolling
Source: Plant Direct. 2020 Oct 30;4(10):e00282. doi: 10.1002/pld3.282 (PMC7598327; doi:10.1002/pld3.282)
Supplement: Supplementary file 1 — Fig S1‐S7 [file PLD3-4-e00282-s001.docx]

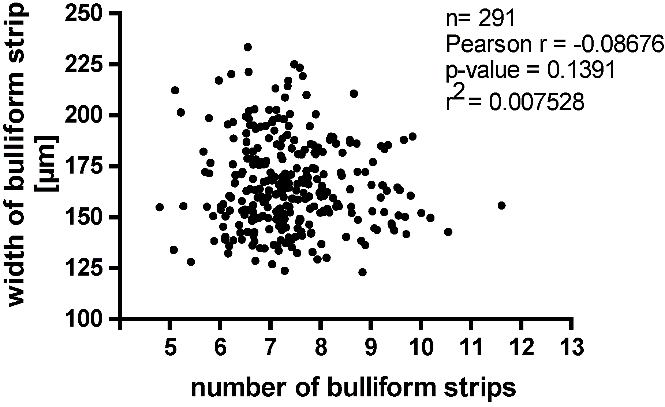


**Supplemental Figure S1. Bulliform strip number and width are not correlated.** Pearson’s correlation analysis of bulliform strip number with width in the population of 291 maize inbred lines analyzed for leaf rolling.


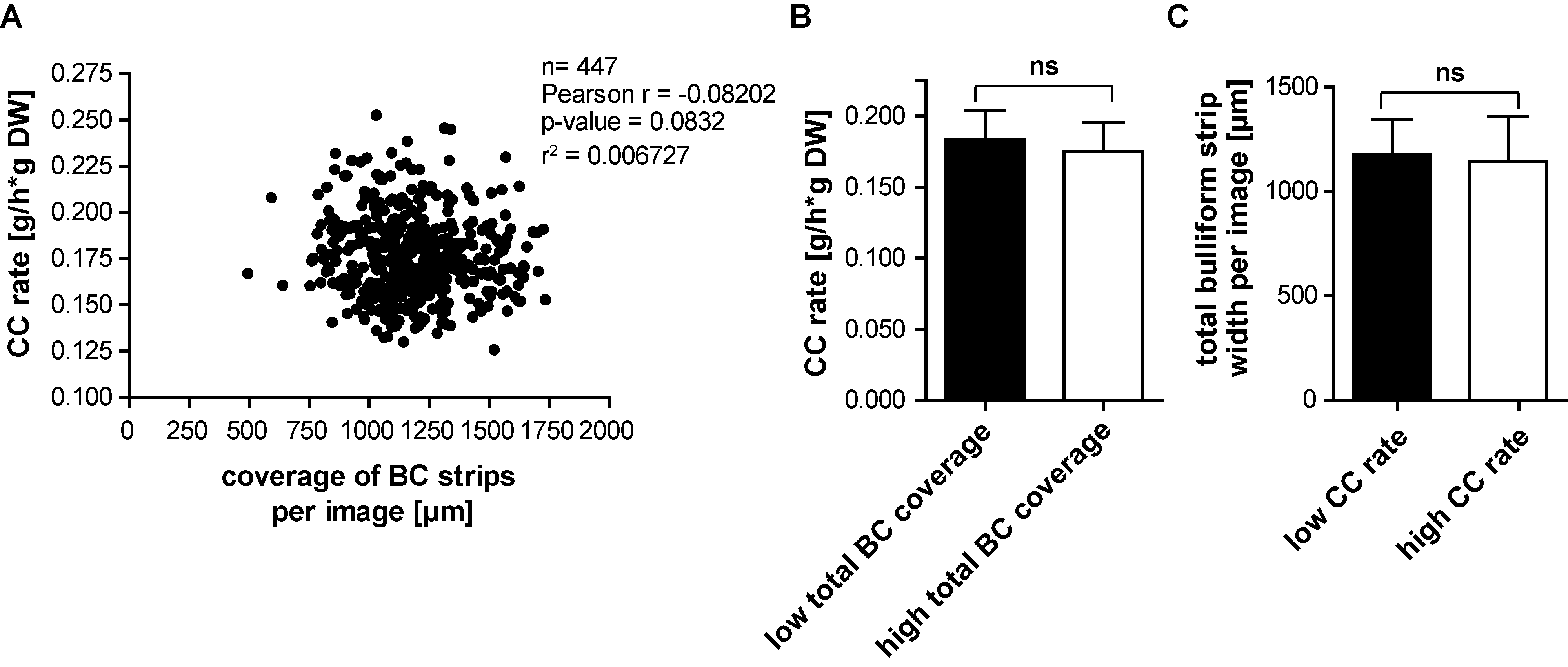


**Supplemental Figure S2: Total BC coverage and cuticular conductance are not correlated in a survey of diverse maize inbred lines.** A) Pearson’s correlation analysis of bulliform strip coverage, i.e. total area occupied by BCs per standard size field of view (Qiao et al., 2019) vs. cuticular conductance g_c_ (Lin et al., 2020) in a collection of 447 maize inbred lines analyzed for both traits. B) Outlier analysis of g_c_ in 30 inbreds with low and 30 with high BC coverage. C) Outlier analysis of BC coverage in 30 lines with low and 30 with high g_c_. Statistical analysis used two‐tailed unpaired Student's *t*‐test.


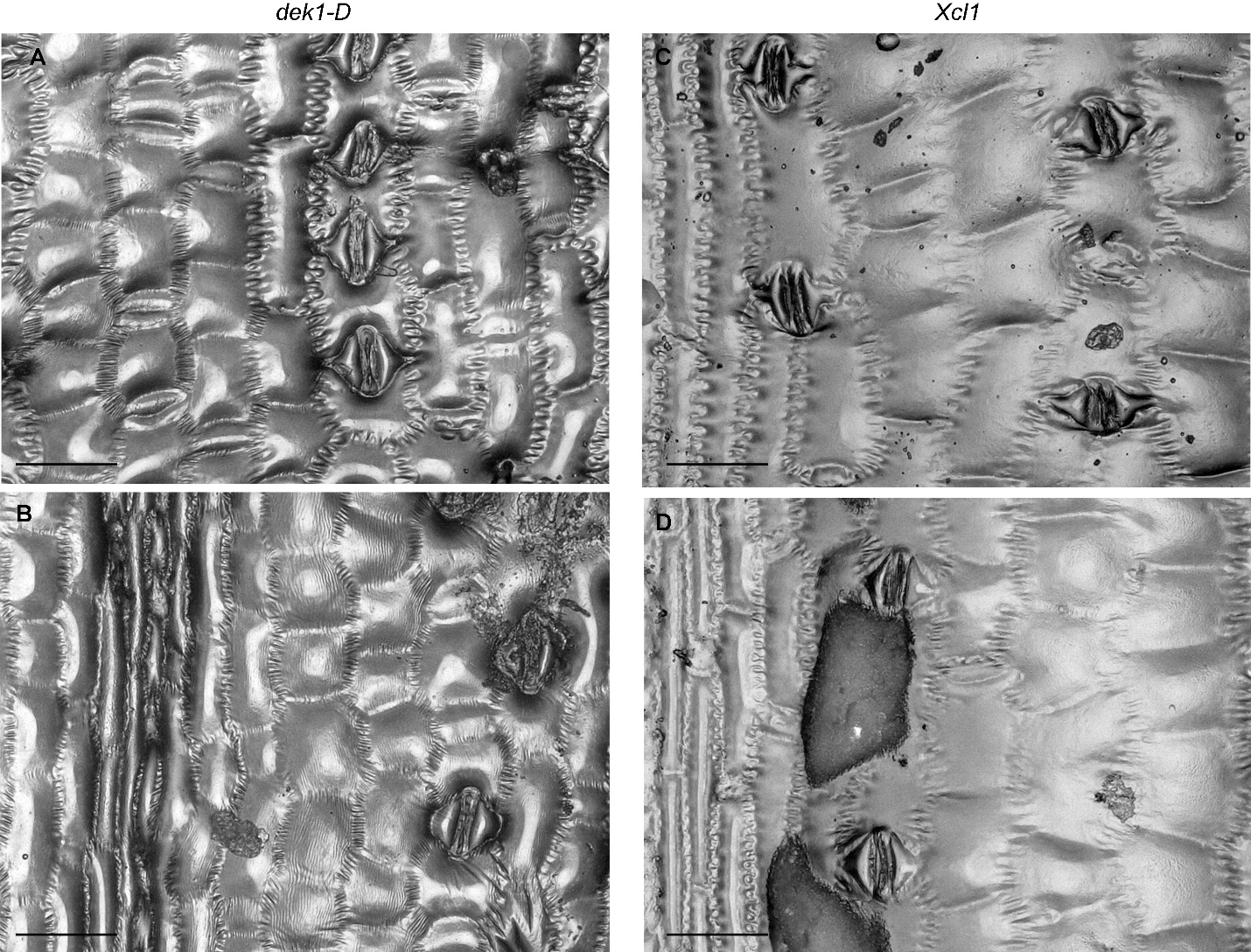


**Supplemental Figure S3: Abaxial BC-like cells in bulliform-enriched mutants do not necessarily have cuticle nanoridges.** Abaxial epidermal glue impressions of bulliform-enriched mutants *dek1-D* (A,B) and *Xcl1* (C,D), two examples per genotype. Scale bar = 50 µm.


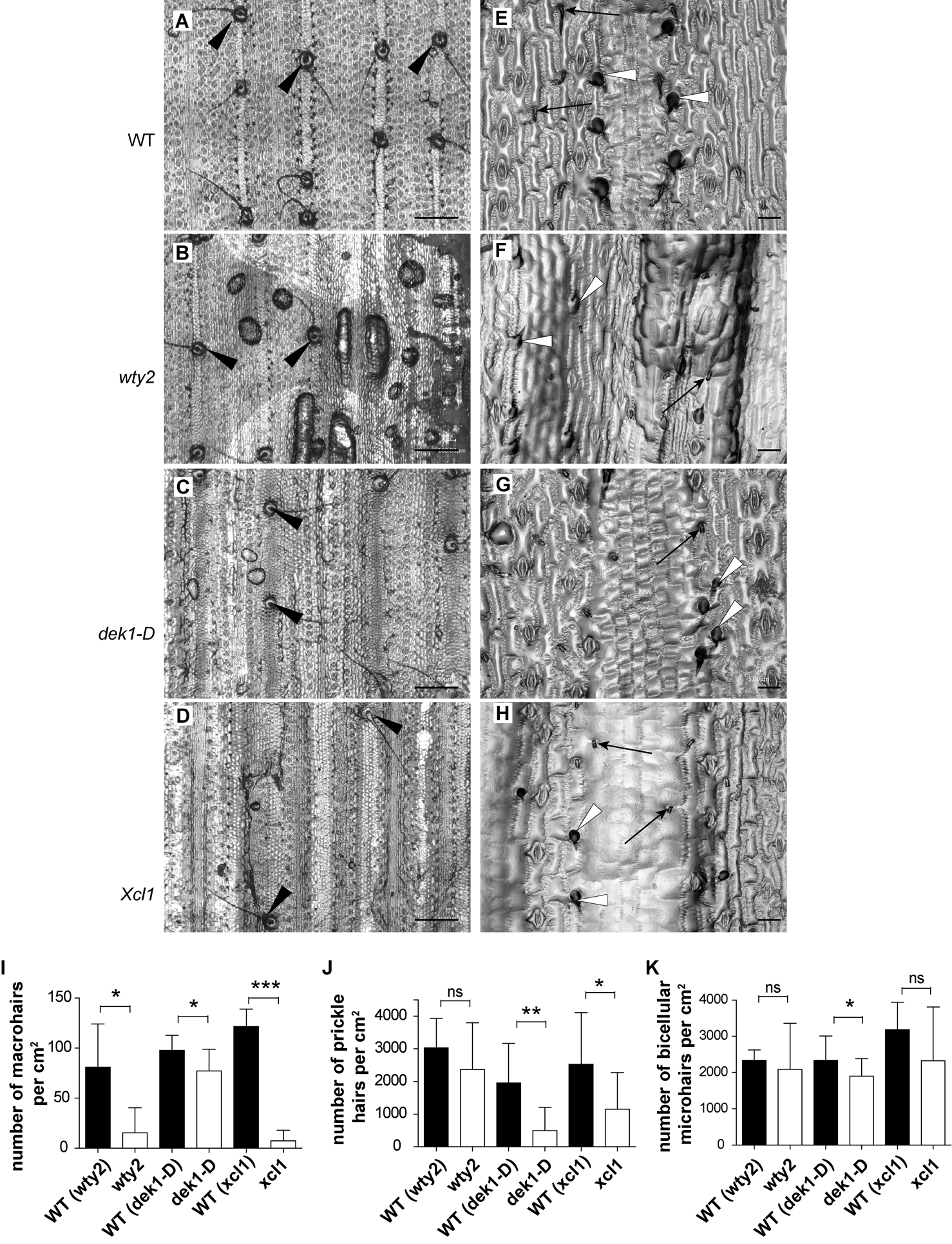


**Supplemental Figure S4: Bulliform-enriched mutants show a reduction in adaxial hair types.** A-H) Epidermal glue impressions of wild-type (A,E) and three bulliform-enriched mutants *wty2* (B,F), *dek1-D* (C,G), and *Xcl1* (D,H) at different magnifications. Black arrowheads indicate macrohairs, white arrowheads show prickle hairs, and black arrows indicate bicellular microhairs, which sometimes appear to be broken off, probably due to handling while creating the glue impressions. I-K) Quantification of the different hair types in the three bulliform-enriched mutants and their corresponding wild-types. Scale bar in A-D = 500 µm, in E-H = 50 µm. Values are given as means ± SD (n = 3 images each of 5 biological replicates per genotype). Statistical analysis used two‐tailed unpaired Student's *t*‐test, with *P < 0.05, **P < 0.01, and ***P < 0.001.


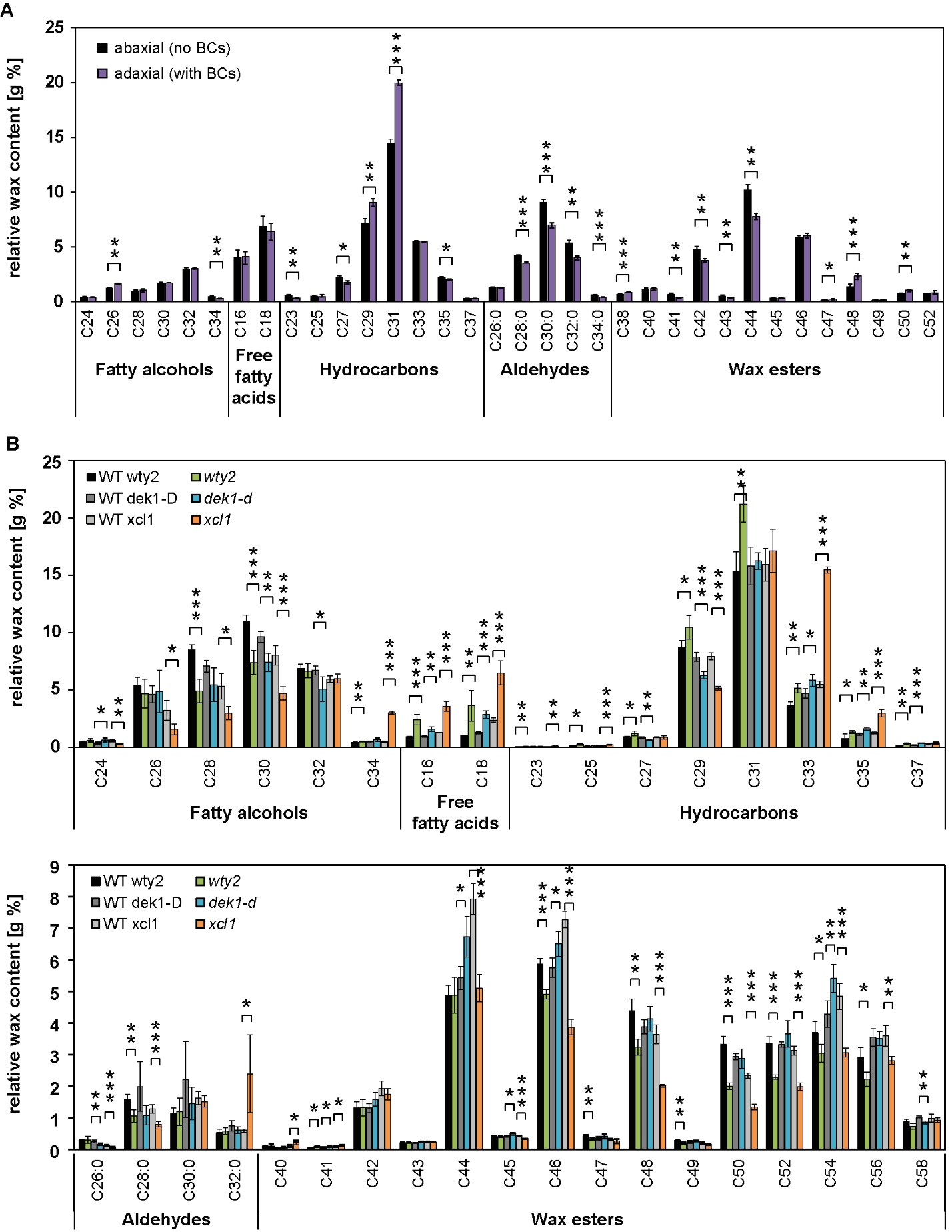


**Supplemental Figure S5: Single compound wax profiles of bulliform-enriched cuticles.** A) Relative content of single wax compounds in adaxial and abaxial tissues after normalization to overall wax load, chloroform-extracted from epidermal peels after enzymatic digestion, measured by GC-MS. B) Relative content of single wax compounds in bulliform-enriched mutants was normalized to overall wax load. Values are given as means ± SD (n = 4 biological replicates per surface/genotype). Statistical analysis used two‐tailed unpaired Student's *t*‐test, with *P < 0.05, **P < 0.01, and ***P < 0.001.


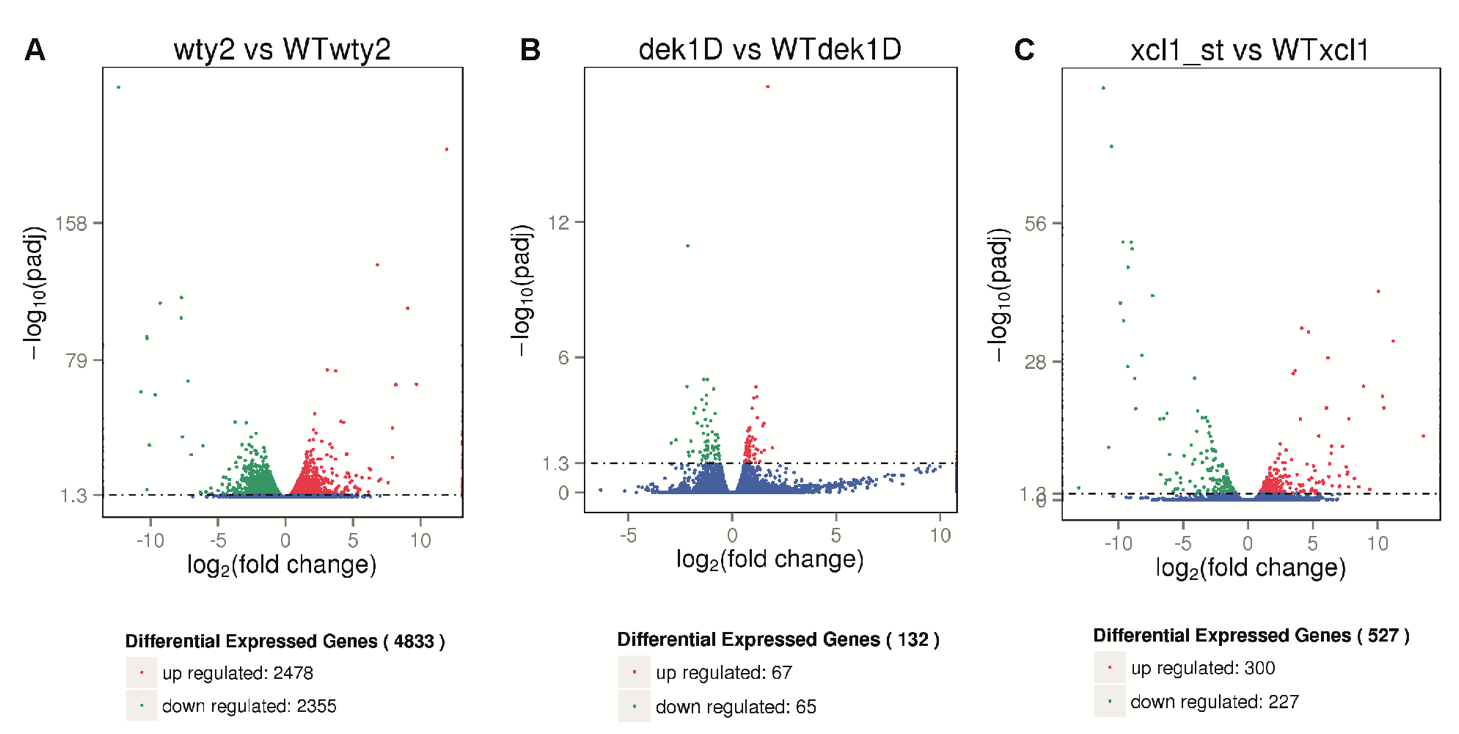


**Supplemental Figure S6: RNAseq analysis of bulliform-enriched mutants.** The cuticle maturation zone of developing adult leaves (10-30 % of leaf length of the maturing leaf at 50 to 60 cm, according to Bourgault et al., 2020) of bulliform mutants and corresponding wild-types was harvested and subjected to gene expression analysis. A-C) Volcano plot showing differentially expressed genes (DEG) for bulliform mutants *wty2* (A), *dek1-D* (B), and *Xcl1* (C) compared to their respective wild-type controls. log2 (fold change) of gene expression is plotted against ‑log10 of adjusted P‑values. red = genes with increased expression in the mutant, green = genes with decreased expression in the mutants.


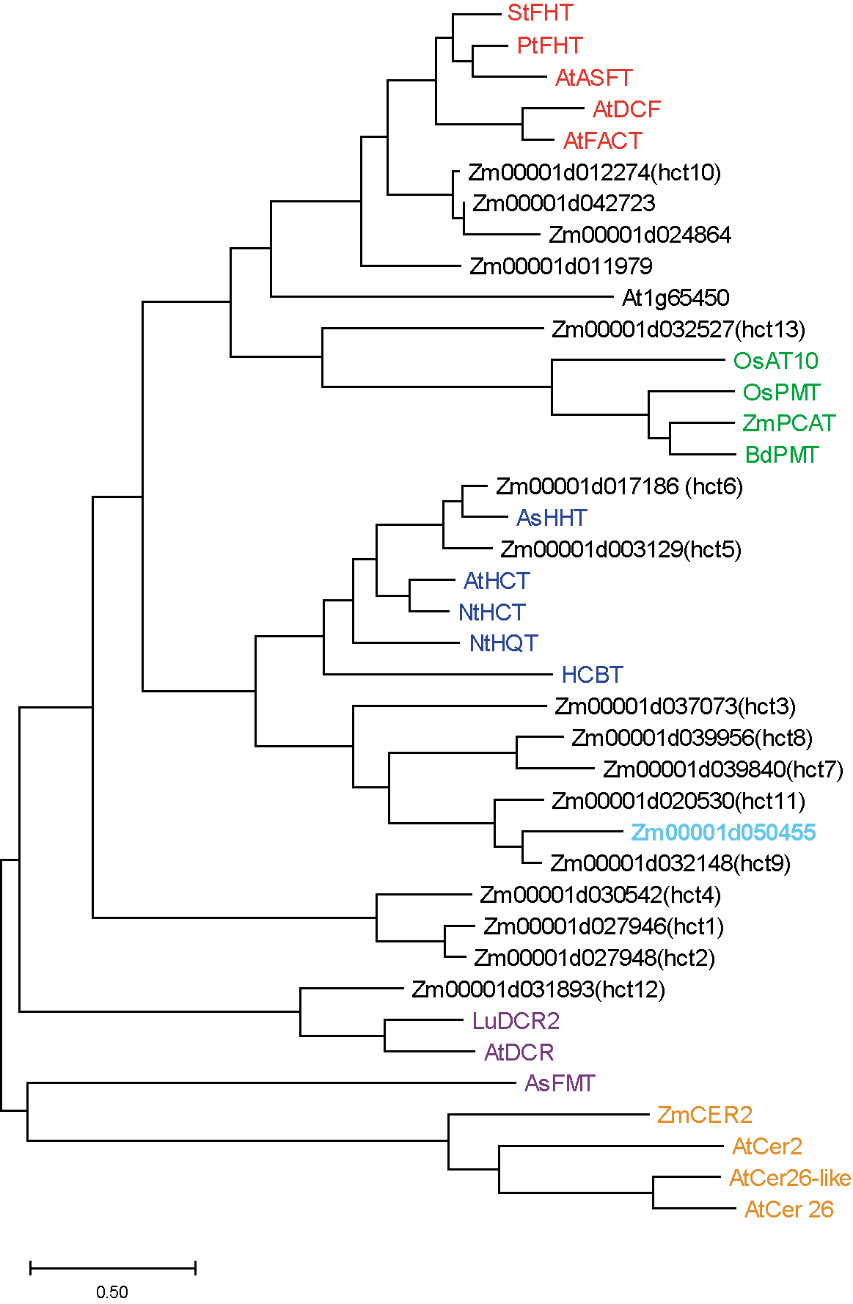


**Supplemental Figure S7: Phylogeny of selected BAHD family acyltransferases with maize candidate genes.** BAHD family enzymes with roles in hydroxycinnamol-CoA transfer are grouped according to Molina and Kosma (2015) and correspond to those related to extracellular lipid biosynthesis (red), cell wall feruloylation/coumaroylation (green) and lignin biosynthesis (blue). The purple clade groups Arabidopsis DCR, which is involved in cutin biosynthesis, and two flax orthologs. The orange clade includes CER enzymes with functions in very long-chain fatty acid biosynthesis. The evolutionary history was inferred by using the Maximum Likelihood method and JTT matrix-based model (Jones et al., 1992). Initial trees for the heuristic search were obtained automatically by applying Neighbor-Join and BioNJ algorithms to a matrix of pairwise distances estimated using a JTT model, and then selecting the topology with superior log likelihood value. The tree is drawn to scale, with branch lengths measured in the number of substitutions per site. This analysis involved 38 amino acid sequences. There were a total of 1085 positions in the final dataset. Evolutionary analyses were conducted in MEGAX (Kumar et al., 2018). The analysis included 21 amino acid sequences of functionally characterized BAHDs from the original tree in Molina and Kosma (2015), and the following 17 maize genes, chosen by sequence homology with Arabidopsis genes in this tree: Zm00001d027946 (hct1), Zm00001d027948 (hct2), Zm00001d037073 (hct3), Zm00001d030542 (hct4), Zm00001d003129 (hct5), Zm00001d017186 (hct6), Zm00001d039840 (hct7), Zm00001d039956 (hct8), Zm00001d032148 (hct9), Zm00001d012274 (hct10), Zm00001d020530 (hct11), Zm00001d031893 (hct12), Zm00001d032527 (hct13), Zm00001d011979, Zm00001d042723, Zm00001d024864, and Zm00001d050455 (light blue, identified DEG in RNAseq of bulliform mutants).

**Supplemental Table S1.** Leaf rolling and bulliform strip patterning data

**Supplemental Table S2.** Natural variation data for BC coverage and cuticular conductance

**Supplemental Table S3.** Genes differentially expressed in *wty2* vs. WTwty2

**Supplemental Table S4.** Genes differentially expressed in *dek1-D* vs. WTdek1-D

**Supplemental Table S5.** Genes differentially expressed in *Xcl1* vs. WTXcl1

**Supplemental Table S6.** Genes differentially expressed in all three bulliform-enriched mutants

**Supplemental Table S7**. TAIR protein BLAST results for Zm00001d008957

**Supplemental Table S8.** TAIR protein BLAST results for Zm00001d050455

**Supplemental Methods:**

*Correlation analysis of bulliform cell architecture and cuticular conductance g_c_:*

Pearson’s correlations between bulliform patterning (number x width of BC strips, Supplemental Table S2, extracted BLUPs for both environments (Maricopa and San Diego) from Qiao et al., 2019) and cuticular conductance g_c_ data from Lin et al. (2020) were analyzed for 447 inbreds in total. Lines with extreme g_c_ (30 highest and 30 lowest) were grouped and additionally graphed for BC architecture independently, as well as lines with extreme BC architecture (30 highest and 30 lowest coverage) were grouped and additionally graphed for their g_c_.

**Supplemental References:**

Bourgault R, Matschi S, Vasquez M, Qiao P, Sonntag A, Charlebois C, Mohammadi M, Scanlon MJ, Smith LG, Molina I (2020) Constructing functional cuticles: analysis of relationships between cuticle lipid composition, ultrastructure and water barrier function in developing adult maize leaves. Ann Bot 125: 79–91

Jones D, Taylor W, Thornton J (1992) The rapid generation of mutation data matrices from protein sequences. Comput Appl Biosci 8: 275–282

Kumar S, Stecher G, Li M, Knyaz C, Tamura K (2018) MEGA X: Molecular Evolutionary Genetics Analysis across Computing Platforms. Mol Biol Evol 35: 1547–1549

Molina I, Kosma D (2015) Role of HXXXD-motif/BAHD acyltransferases in the biosynthesis of extracellular lipids. Plant Cell Rep 34: 587–601
